# On Medical Tourism Overseas: Ethical Analysis of the Duties of NHS Doctors in Managing the Negative Health Consequences of Accessing Medical Treatments Abroad

**DOI:** 10.1111/jep.14300

**Published:** 2025-01-08

**Authors:** Richard C. Armitage

**Affiliations:** ^1^ Academic Unit of Population and Lifespan Sciences School of Medicine University of Nottingham Nottingham UK

## Abstract

**Introduction:**

An increasing number of UK residents are travelling overseas to access medical treatments, the negative health consequences of which are largely managed by NHS doctors.

**Methods:**

This paper performs an ethical analysis, using the ethical framework of principlism, of the duties of NHS doctors in managing these negative health consequences of medical tourism overseas.

**Findings:**

While the doctor's duty to respect patient autonomy contains a negative duty to not interfere with their choice to access medical treatment overseas, it also contains a positive duty to ensure this choice is informed. This requires those considering medical tourism overseas to be counselled on the risks. This should take place directly by counselling, and indirectly through public health messaging. Beneficence requires the doctor to promote the patient's health, therefore obligating them to treat complications of medical tourism overseas, to intervene if poor cosmetic outcomes negatively impact the patient's mental health, and to refer the patient if the necessary aftercare is insufficiently or entirely unavailable on the NHS. Beneficence also requires doctors to remove harm, meaning they must counsel patients about the risks of medical tourism overseas to minimise the risk of negative health consequences. Justice requires NHS doctors to care for patients according to their clinical needs regardless of how that need has arisen, including the negative health consequences of medical tourism abroad, and requires NHS doctors to minimise these negative health consequences to minimise the scarce resources allocated to addressing them. The duty of non‐maleficence is not relevant in this context.

**Conclusion:**

Amongst other requirements, this paper finds that NHS doctors must counsel those considering medical tourism overseas on the risks of doing so, and existing efforts to do so should be increased to reflect the increasing prevalence of medical tourism overseas by UK residents and the associated negative health consequences.

## 1 Introduction

The National Health Service (NHS) is the publicly‐funded single‐payer healthcare system in the United Kingdom (UK). It is funded by the government from general taxation and provides comprehensive, universal and free at the point of delivery healthcare for UK residents on the basis of clinical need. The NHS is currently operating under substantial and growing pressures, with declining general practitioner (GP) numbers and rising patient demand in primary care [1], and longer waits for emergency care and record numbers of patients awaiting outpatient treatment and elective (planned) surgery in secondary care [2]. The NHS does not provide cosmetic treatment beyond that which addresses the consequences of underlying illness. Alongside the NHS exists a private healthcare sector in the UK that provides elective medical services including cosmetic treatments that are not available on the NHS. Private providers are regulated by the relevant body in each part of the UK, such as the Care Quality Commission in England.

### 1.1 Medical Tourism Overseas

Medical tourism is the practice of travelling to another country to access medical treatment that is paid for by the patient out of pocket [3]. In recent years there has been an increase in the number of patients from more developed countries travelling to less developed countries for the purpose of medical tourism. This has formed a ‘medical tourism industry’ that is largely driven by the lower cost of medical treatments in less developed countries, the falling cost of air travel, the internet‐mediated marketing of these possibilities [4] and the availability of ‘medical package holidays’ [5]. In the last two decades, a large and growing number of UK residents—around 50,000 in 2010 [6], 152,000 in 2018 [7], 234,000 in 2021 [8], 348,000 in 2022 [9], and 431,000 in 2023 [10]—have travelled overseas for medical tourism. It is recognised that the standards of healthcare facilities and medical treatments, and the rigour of regulatory oversight, vary widely across the globe. This, in addition to predatory recruitment tactics, inadequate aftercare and the misleading reassurance of ‘medical package holidays’, has prompted concerns from various UK governmental and medical authorities over the dangers posed to UK residents from medical tourism overseas.

For example, the UK's Foreign, Commonwealth & Development Office discusses medical tourism in Turkey, noting that ‘we are aware of over 25 British nationals who have died in Turkey since January 2019 following medical procedures’ [11]. The British Association of Aesthetic Plastic Surgeons advises against traveling overseas for surgery of any kind (not only cosmetic), and states that ‘patients who opt for medical tourism are risking their health’ [12]. The British Association of Plastic, Reconstructive and Aesthetic Surgeons recognises the substantial risk of undergoing cosmetic surgery overseas, and notes that it has ‘noticed an increasing number of patients turning to the NHS with problems following cosmetic surgery abroad’ [13]. The Human Fertilisation and Embryology Authority highlights that many countries do not regulate fertility treatment as strictly as the UK [14], while the British Dental Association recognises that ‘many UK dentists are now picking up the pieces’ following complications of dental treatment overseas [15]. The British Obesity and Metabolic Surgery Society is becoming ‘increasingly concerned by the number of patients presenting with the complications of surgical procedures performed outside the UK’, and notes that ‘There is often an assumption that the NHS will sort out any issues once the patient returns home’ [16]. Despite these concerns, an increasing number of UK residents choose to access medical treatment overseas. Accordingly, the NHS has issued advice to patients considering it [17, 18], including how to recognise potential warning signs, and additional advice specifically for those considering cosmetic surgery overseas [5].

The negative health consequences of medical tourism overseas, and their costs to the NHS, have been recognised for over a decade [19, 20], and the tensions that medical tourism overseas places on the GP–patient relationship have been noted in a comparable setting [21]. Yet, the duties of NHS doctors in managing these consequences have as yet not been examined. The publicly funded nature of the NHS, combined with the growing concerns from UK governmental and medical authorities over the dangers posed to UK residents from medical tourism overseas, raises the question of how NHS doctors should manage the negative health consequences of medical tourism overseas? This paper shall attempt to answer this by examining the duties of NHS doctors in managing these negative health consequences through an ethical analysis, specifically using the ethical framework of principlism [22], which NHS doctors often use to navigate ethical challenges in their clinical practice. It shall consider this question from an exclusively ethical perspective, and will not attempt a legal analysis. It will outline the three major reasons for why UK residents travel overseas to access medical treatment, before describing the three major categories of negative health consequences that medical tourism overseas generates. It will then introduce principlism, before considering the duties of beneficence, respect for autonomy, non‐maleficence and justice in the context of the negative health consequences of medical tourism overseas.

### 1.2 Reasons for Medical Tourism Overseas

Broadly, there are three main reasons why UK residents travel overseas to access medical treatment. The first is for treatments that are unavailable on the NHS. These can be divided into three broad categories. First, treatments that are purely cosmetic in nature, and therefore do not address a health or medical need, but rather an aesthetic aspiration. Some cosmetic surgeries—such as cosmetic dentistry, breast augmentation, liposuction, tummy tuck, face lift and eye lid surgery—are available in the UK's private healthcare sector, but they are often available overseas at significantly lower cost. Second, treatments that are unproven, unsafe or experimental. These are neither available on the NHS nor in the UK's private healthcare sector. Thirdly, treatments that are both effective and safe, but do not reach the required threshold of cost‐effectiveness to be made available on the NHS. For example, the UK's National Institute for Health and Care Excellence recently found the incremental cost‐effectiveness ratio of the use of trastuzumab deruxtecan for treating HER2‐low metastatic or unresectable breast cancer after chemotherapy to be above £30,000 per quality‐adjusted life year gained, which is the upper end of the range normally considered a cost‐effective use of NHS resources [23].

The second reason is for treatments that are available on the NHS, but that the patient is not eligible to receive. For example, patients can only be considered for bariatric surgery on the NHS if they have a body mass index of ≥ 40 or 35–40 kg/m^2^ with a significant health condition that could be improved by weight loss [24]. Simultaneously, private healthcare providers in the UK generally adhere to the same eligibility criteria as the NHS. However, some overseas providers offer bariatric surgery to patients who are not eligible to receive it in the UK, and often at a substantially lower cost than the same surgery provided in the UK's private healthcare sector (to date, at least one UK resident has died due to a complication of bariatric surgery overseas that they were not eligible to receive in the NHS) [25].

The third reason is for treatments that are available on the NHS and that the patient is eligible to receive, but the waiting time is intolerably long. Examples include joint replacement surgery, fertility treatment, bariatric surgery and gender reassignment medication and surgery. While these treatments might also be available in the UK's private healthcare sector with much shorter waiting times, these treatments are often available with a similarly short waiting time yet at significantly lower cost overseas.

### 1.3 Negative Health Consequences of Medical Tourism Overseas

There are three major categories of negative health consequences that medical tourism overseas generates. The first is complications (including life‐threatening emergencies) that are directly related to the medical treatment received overseas [26]. Examples include infectious complications (including wound infection, abscesses/collections and sepsis, potentially with multidrug‐resistant organisms) [27, 28], bleeding and haematoma [29], wound dehiscence [30], severe dehydration, nerve palsy [31], bowel perforation and bowel obstruction [32]. These complications might necessitate a prolonged inpatient stay, further surgery and admission to the Intensive Care Unit (ICU) [32] and might lead to death [31]. Some might present months after the medical treatment overseas was undertaken [32], such as pain, implant rupture and capsular contracture, which might require further surgical intervention [29].

The second category is poor cosmetic outcomes as perceived by the patient [13]. These results do not amount to complications that threaten the individual's health, but constitute aesthetic outcomes that are deemed unfavourable by the patient. These negative health consequences mostly arise following cosmetic surgeries.

The third category is if the necessary aftercare that is required following medical treatment overseas is insufficiently available or entirely unavailable in the UK. Examples include medical treatments or surgical procedures that are not available on the NHS, blood monitoring after bariatric surgery and continued prescribing of hormonal therapies for gender reassignment. If medical treatments have been initiated or surgical procedures carried out overseas that are unavailable in the NHS, the NHS is unlikely to contain the required professional skillset to continue the medical treatment safely or follow‐up the surgical procedure appropriately (and, if unproven, unsafe or experimental treatments have been initiated or performed overseas, expertise with regard to these are by definition unavailable in the UK). Similarly, if treatments that are available on the NHS have been initiated overseas in patients who are not eligible to receive them on the NHS, NHS professionals will only be skilled and experienced in providing the necessary aftercare for these treatments in populations who are eligible to receive them. Additionally, when surgeries have taken place overseas on patients who are eligible to receive them in the NHS but who have not first undergone the necessary preparatory psychological therapy (such as gender reassignment surgery), access to the relevant post‐surgical psychological therapies might not be immediately available in the UK. Furthermore, discrepancies might arise between products used in the NHS and in the overseas healthcare facilities where treatments take place, such as in pharmaceutical products (medications, doses, and formulations) and medical devices (such as gastric bands, breast implants and prosthetic joints), which creates challenges to the continuation of specific medications and the provision of appropriate aftercare in the UK.

## 2 Methods

Principlism is the normative ethical framework of professional ethics that was devised by Beauchamp and Childress [22]. This framework, which was designed to aid ethical decision‐making in healthcare contexts, comprises four basic and universal ethical principles that state prima facie moral obligations that are equally important for doctors in the provision of patient care. The Four Principles are the duties of respect for autonomy, beneficence, justice and non‐maleficence. Since their introduction, the Four Principles have been the primary method for the teaching and evaluation of medical ethical dilemmas in healthcare contexts and [33], due to their strong support [34, 35, 36, 37], are still widely used today.

An ethical analysis of the duties of NHS doctors in managing the negative health consequences of medical tourism overseas in UK residents shall now be conducted using the principlism framework. This analysis will only address the negative health consequences of medical tourism overseas. It will not, therefore, examine the potential positive health consequences of medical tourism overseas, which might include UK residents who receive treatments overseas that are both effective and safe but are not available on the NHS due to their insufficient cost effectiveness, and UK residents who receive treatments overseas that they are eligible to receive on the NHS but who would have to endure long waiting lists to receive them at home. Such patients demonstrate the potential advantages of medical tourism overseas (both to the patients themselves and to the NHS), but this is not the focus of this paper. Instead, this paper will focus exclusively on the duties of NHS doctors in managing the *negative* health consequences of medical tourism overseas.

It is also recognised that medical tourism is a bidirectional activity, and the UK enjoys substantial economic benefits through its private healthcare sector's provision of treatments to in‐bound medical tourists from overseas (some 34,000 overseas residents visited the UK for medical treatment in 2021) [8]. In addition, the local economies of overseas countries which provide treatments for medical tourists from the UK also greatly benefit from these activities. This paper, however, will not focus on the ethical implications of these factors. Nor will it examine the ethical duties that overseas doctors might owe to the UK medical tourists that they treat, or the ethical implications of overseas doctors (who are often located in less developed countries than the UK) treating UK medical tourists rather than their own populations. Instead, the paper will only focus on the duties of NHS doctors in managing the negative health consequences of medical tourism overseas in UK residents.

## 3 Findings

### 3.1 Autonomy

Autonomy is the principle that individuals have the right to make decisions, hold views and undertake actions based on their personal views and values. Broadly speaking, a person is autonomous if they govern their own decisions and actions. Accordingly, autonomy requires the doctor to respect the patient's capacity for self‐determination, and their capacity to make independent decisions about their life in the absence of undue pressure, solicitation or coercion. Fundamentally, a failure to respect a patient's autonomy involves interfering with their capacity to make autonomous choices, or interference with the patient's opportunity to act upon those choices, or both.

Due to their obligation to respect patient autonomy, doctors hold a prima facie duty to not interfere with the decision of a UK resident to travel overseas to access medical treatment. This would discharge the doctor's ‘negative’ duty to autonomy, which requires them to avoid performing acts that would interfere with the patient's autonomy such as influencing the patient through manipulation, deception, coercion, threats or undue incentives. In this way, the doctor respects the patient's decisions about the actions they take in their own life in a similar manner to how the doctor respects the patient's decisions about other lawful yet risky (in terms of health) actions such as consuming alcohol, smoking cigarettes and engaging in risky sexual behaviour.

However, in addition to the doctor's negative duty is their ‘positive’ duty to autonomy, which requires the doctor to perform certain acts such as providing opportunities for patients to make their own choices, enabling patients to act on their own choices, and providing patients with understandable information regarding their health and medical treatment. For patients to make truly autonomous choices their consent to make those choices must be valid, which requires them to have access to and understand the relevant information pertaining to each choice. In the context of alcohol consumption, cigarette smoking and risky sexual behaviour, doctors have a duty to inform patients of the health risks associated with these choices, which they perform directly by counselling patients in consultations and indirectly in the wider landscape of public health interventions (including messaging, and harm reduction products and services), advertising standards and national legislation. Similarly, the doctor's positive duty to autonomy requires them to inform UK residents who are considering travelling overseas to access medical treatment of the risk of negative health consequences of doing so, and about the extent to which NHS doctors would manage those that subsequently occur. This ought to take place directly by counselling such patients in consultations, and indirectly through public health messaging, to maximise the availability and comprehension of relevant information with which patients can make informed, and thus autonomous, choices about medical tourism overseas.

While efforts are already being made to achieve this by various UK governmental and medical authorities (see Section 1), more aggressive messaging and educating doctors on this matter to improve the quality of their counselling, is needed to reflect the increasing prevalence of both medical tourism overseas by UK residents and the associated negative health consequences. It is noted, however, that while NHS doctors are deeply familiar with the health risks associated with, for example, alcohol consumption, cigarette smoking, and risky sexual behaviour (since the consequences of these behaviours are widely included in the UK's medical education curricula and competencies in their identification, minimisation and management are required by all doctors who practice in the NHS), their familiarity with treatments that take place overseas—especially those that are unavailable on the NHS, including those that are purely cosmetic in nature, and treatments that are unproven, unsafe or experimental—is limited.

Accordingly, their ability to inform UK residents who are considering travelling overseas to access medical treatment about that medical treatment is less than complete. In these cases, the NHS doctor's duty to autonomy requires them to inform the patient of their limited familiarity with the proposed treatment, and of the lack of sound evidence base for treatments that are unproven, unsafe or experimental. In an adjacent but meaningfully different situation, a patient may be considering accessing treatment overseas that is unavailable on the NHS due to insufficient cost‐effectiveness despite being both clinically effective and safe. In this case, the NHS doctor's duty to autonomy requires them to inform the patient of the existing evidence base for the treatment's clinical utility (which is favourable), and explain to the patient that, if the treatment were cheaper, it would likely be available on the NHS. Since the health consequences of this form of medical tourism overseas are expected to be positive, it is not the direct focus of this paper (which examines the negative health consequences), yet it is relevant to the discussion on autonomy.

### 3.2 Beneficence

Beneficence requires doctors to act for the benefit of the patient, such as preventing or removing harm, or the active promotion of some good, such as health. In the context of a UK resident who experiences negative health consequences of the medical treatment they accessed overseas, what the duty of beneficence requires of doctors in the UK is determined by the category of negative health consequences being experienced.

In cases of the first category—complications of the medical treatment received overseas, including life‐threatening emergencies, infectious complications, bleeding, wound dehiscence, severe dehydration and bowel perforation—beneficence requires the doctor to treat the complication in an attempt to restore the patient's health to the greatest degree possible. The nature of this treatment is determined by the kind of complication, the most severe of which might require immediate and intense intervention such as the administration of antimicrobials, inpatient stay, further surgery and ICU admission, while the less severe complications might require less urgent treatment such as conservative management and subsequent elective surgery. This is required of doctors to promote the patient's health, which is severely and directly diminished by the complications of the medical treatment received overseas. Additionally, since beneficence also requires doctors to prevent harm, they must inform UK residents of the risks of negative health consequences of accessing medical treatment overseas—specifically, complications.

In cases of the second category—poor cosmetic outcomes that do not amount to complications that diminish the patient's health—the duty of beneficence does not necessarily require any intervention by the doctor. Only if the cosmetic outcome is deemed so unfavourable by the individual that it induces sufficient and enduring psychological distress to negatively impact their mental health would beneficence require intervention. Initially, however, such patients should directly contact and raise their concerns with the provider of the care they are unsatisfied with. While the more reputable overseas providers make such channels of communication available to their patients and might have UK representatives available for patients to access following their care overseas, less reputable providers might not offer such reassurances [13]. If subsequent surgery is required to improve the cosmetic outcome, the patient might not have the financial means to do so, either overseas or in the UK's private healthcare sector. If the patient is unable to have poor cosmetic outcomes improved, and this is found to have consequential negative impacts on their mental health, the duty to beneficence would require the doctor to intervene through the provision of psychological support services, and potentially subsequent surgery (while the NHS does not perform cosmetic surgeries, it might have an ethical duty to fund the relevant surgery in the UK's private healthcare sector to promote the mental health of the patient). Here, however, beneficence clashes with the duty to justice, which requires NHS doctors to fairly distribute the NHS's scarce resources amongst patients, and shall be discussed later. Furthermore, since beneficence also requires doctors to prevent harm, they must inform UK residents of the risks of negative health consequences of accessing medical treatment overseas—specifically, the potential mental health impacts of poor cosmetic outcomes.

In cases of the third category—if the necessary aftercare that is required following medical treatment overseas is insufficiently available or entirely unavailable in the UK—beneficence requires doctors to promote the patient's health to the extent that it is possible. This generally requires the patient to be referred to colleagues with the closest relevant expertise, since this kind of clinician's skillset has the greatest probability of promoting the patient's health. For example, if medical treatments have been initiated or surgical procedures carried out overseas that are unavailable in the NHS (particularly those that are unproven, unsafe or experimental), the doctor must refer the patient to colleagues with the closest relevant skillset (e.g., if a form of bariatric surgery, i.e., not available in the UK is performed overseas, the patient should be referred to a bariatric surgeon in the United Kingdom). Similarly, if treatments that are available on the NHS have been initiated overseas in patients who are not eligible to receive them on the NHS, the patient should be preferred to colleagues who specialise in those treatments (albeit in populations who are eligible to receive them). Likewise, if medical treatments have taken place overseas in patients who are eligible to receive them in the NHS but who have not first undergone the necessary preparatory psychological therapy, the patient should be urgently referred for relevant psychological aftercare. Finally, if discrepancies arise between the products used in the NHS and those in the overseas healthcare facilities in which treatments take place (such as pharmaceutical products and medical devices), the patient should be referred to colleagues with the closest relevant expertise. In addition, since beneficence also requires doctors to prevent harm, they must inform UK residents of the risks of negative health consequences of accessing medical treatment overseas—specifically, if the necessary aftercare is insufficiently available or entirely unavailable in the United Kingdom.

### 3.3 Justice

The principle of justice requires doctors to ensure that the benefits and costs of actions are fairly distributed between patients. Since the NHS is publicly funded from general taxation, justice requires NHS doctors to distribute the NHS's scarce resources between UK residents fairly. What ‘fairly’ amounts to, however, is subject to various interpretations, yet it is most commonly considered to constitute an aspiration of health equity rather than one of health equality. One of the seven key principles guide the NHS in all it does is that ‘access to NHS services is based on clinical need, not an individual's ability to pay’ [38], and no consideration is made of the means by which that clinical need has arisen. As such, the consumption of scarce NHS resources to address the negative health consequences of an individual's decision to consume alcohol, smoke cigarettes, misuse illicit substances or engage in risky sexual behaviour is similar in kind to the use of those resources to address the negative health consequences of an individual's decision to access medical treatment overseas.

However, the financial cost to the NHS of treating the negative health consequences of medical tourism overseas amounts to many thousands of pounds per patient [39] (comparable costs are reported in other developed countries) [40, 41, 42]. These costs are in addition to the professional time that doctors and other healthcare professionals must use to treat these negative health consequences. Furthermore, many of those that do are eligible to receive their medical treatment on the NHS but wish to avoid waiting, yet the cost to the NHS of addressing the complications of their overseas treatment exceeds that of the treatment had it taken place on the NHS [43]. Additionally, treating these negative health consequences redirects substantial NHS resources that would otherwise be utilised to address other health problems. This further increases the profound pressures under which the NHS is currently functioning. Furthermore, a cross‐border economic injustice occurs, as the UK tax payer realises the financial losses of the negative health consequences of medical tourism overseas, while the financial gains are realised by the countries in which these treatments occur.

Accordingly, distributing scarce shared resources in this manner might be considered unfair, since accessing medical treatment overseas is largely unnecessary (such as when it is available on the NHS) or optional (such as when it is cosmetic). Yet, there is no meaningful moral difference for a patient who engages in other risky behaviours—such as alcohol consumption, cigarette smoking, illicit drug use, or even horse riding or rock climbing—which are arguably similarly unnecessary and optional pursuits. In all cases, justice requires that patients receive medical care on the basis of their clinical need, regardless of how that need has arisen, meaning the NHS doctor's duty of justice requires them to provide the necessary care for patients experiencing the negative health consequences of medical tourism overseas just as they are required to care for those experiencing the negative health consequences of any other decision.

Simultaneously, given the scarce nature of NHS resources, justice requires NHS doctors to act to minimise the negative health consequences of medical tourism overseas to minimise the resources allocated to addressing them. As such, this further requires NHS doctors to counsel those considering medical tourism overseas on the risks of doing so in a similar manner to the requirement of respect for autonomy.

### 3.4 Non‐Maleficence

Non‐maleficence requires doctors, through their medical interventions, to avoid causing intentional harm to patients. Since no doctor in the performs the medical intervention that a UK resident travels overseas to access, non‐maleficence is not relevant in this context. Denying patients access to treatments that they are not eligible to receive in the United Kingdom does not violate the duty of non‐maleficence because their ineligibility is founded on the evidence base that determines those who do not stand to benefit from the treatment as ineligible to receive it.

## 4 Conclusion

An increasing number of UK residents are travelling overseas to access medical treatments, the negative health consequences of which are largely managed by NHS doctors. While the doctor's duty to respect patient autonomy contains a negative duty to not interfere with their choice to do this, it also contains a positive duty to ensure this choice is informed. This requires those considering medical tourism overseas to be counselled on the risks, and about how NHS doctors would manage any negative health consequences. This should take place directly by counselling, and indirectly through public health messaging. Given the increasing prevalence of medical tourism overseas and the associated negative health consequences, existing efforts to achieve this should be increased through more aggressive messaging and educating doctors on this matter. Beneficence requires the doctor to promote the patient's health, and therefore obligates them to treat complications of medical tourism overseas, to intervene if poor cosmetic outcomes negatively impact the patient's mental health, and to refer the patient to colleagues with the closest relevant expertise if the necessary aftercare is insufficiently available or entirely unavailable on the NHS. Beneficence also requires doctors to remove harm, meaning they must counsel patients about the risks of medical tourism overseas to minimise the risk of negative health consequences occurring. The principle of justice requires NHS doctors to care for patients according to their clinical needs regardless of how that need has arisen, including patients experiencing the negative health consequences of medical tourism abroad. Simultaneously, due to the scarce nature of NHS resources, justice requires NHS doctors to act to minimise the negative health consequences of medical tourism overseas to minimise the resources allocated to addressing them. This further requires NHS doctors to counsel those considering medical tourism overseas on the risks of doing so. The duty of non‐maleficence is not relevant in this context.

## Conflicts of Interest

The author declares no conflicts of interest.

## Data Availability

The author has nothing to report.
